# Supplementary material for: Pyruvate Dehydrogenase Kinase 1 inhibition mediated oxidative phosphorylation enhancement in cartilage promotes osteoarthritis progression
Source: BMC Musculoskelet Disord. 2023 Jul 20;24:597. doi: 10.1186/s12891-023-06585-6 (PMC10357736; doi:10.1186/s12891-023-06585-6)
Supplement: Supplementary file 1 — Additional file 1. [file 12891_2023_6585_MOESM1_ESM.zip › MF.Gsea.1653627316242/GOMF_C_ACYLTRANSFERASE_ACTIVITY.html]

Details for gene set GOMF\_C\_ACYLTRANSFERASE\_ACTIVITY[GSEA]

|  || Dataset | 55457\_collapsed\_to\_symbols.cls.cls #OA\_versus\_normal.cls.cls #OA\_versus\_normal\_repos |
| Phenotype | cls.cls#OA\_versus\_normal\_repos |
| Upregulated in class | OA |
| GeneSet | GOMF\_C\_ACYLTRANSFERASE\_ACTIVITY |
| Enrichment Score (ES) | 0.47108263 |
| Normalized Enrichment Score (NES) | 1.4345428 |
| Nominal p-value | 0.09070295 |
| FDR q-value | 0.28009295 |
| FWER p-Value | 1.0 |
Table: GSEA Results Summary

  

Fig 1: Enrichment plot: GOMF\_C\_ACYLTRANSFERASE\_ACTIVITY      
 Profile of the Running ES Score & Positions of GeneSet Members on the Rank Ordered List

  

| SYMBOL | TITLE | RANK IN GENE LIST | RANK METRIC SCORE | RUNNING ES | CORE ENRICHMENT || 1 | SCP2 | sterol carrier protein 2 [Source:HGNC Symbol;Acc:HGNC:10606] | 105 | 0.905 | 0.1758 | Yes |
| 2 | HADHB | hydroxyacyl-CoA dehydrogenase trifunctional multienzyme complex subunit beta [Source:HGNC Symbol;Acc:HGNC:4803] | 675 | 0.599 | 0.2535 | Yes |
| 3 | ACAT1 | acetyl-CoA acetyltransferase 1 [Source:HGNC Symbol;Acc:HGNC:93] | 1017 | 0.513 | 0.3315 | Yes |
| 4 | GCAT | glycine C-acetyltransferase [Source:HGNC Symbol;Acc:HGNC:4188] | 1095 | 0.497 | 0.4266 | Yes |
| 5 | ACSM3 | acyl-CoA synthetase medium chain family member 3 [Source:HGNC Symbol;Acc:HGNC:10522] | 1835 | 0.380 | 0.4465 | Yes |
| 6 | ACAA2 | acetyl-CoA acyltransferase 2 [Source:HGNC Symbol;Acc:HGNC:83] | 2779 | 0.272 | 0.4287 | Yes |
| 7 | SPTLC1 | serine palmitoyltransferase long chain base subunit 1 [Source:HGNC Symbol;Acc:HGNC:11277] | 2913 | 0.259 | 0.4711 | Yes |
| 8 | ACSM2A | acyl-CoA synthetase medium chain family member 2A [Source:HGNC Symbol;Acc:HGNC:32017] | 4080 | 0.148 | 0.4107 | No |
| 9 | HADHA | hydroxyacyl-CoA dehydrogenase trifunctional multienzyme complex subunit alpha [Source:HGNC Symbol;Acc:HGNC:4801] | 4163 | 0.141 | 0.4330 | No |
| 10 | ACSM5 | acyl-CoA synthetase medium chain family member 5 [Source:HGNC Symbol;Acc:HGNC:26060] | 4976 | 0.074 | 0.3851 | No |
| 11 | ACAA1 | acetyl-CoA acyltransferase 1 [Source:HGNC Symbol;Acc:HGNC:82] | 5175 | 0.058 | 0.3816 | No |
| 12 | ACAT2 | acetyl-CoA acetyltransferase 2 [Source:HGNC Symbol;Acc:HGNC:94] | 6291 | -0.027 | 0.3004 | No |
| 13 | SPTSSA | serine palmitoyltransferase small subunit A [Source:HGNC Symbol;Acc:HGNC:20361] | 7346 | -0.106 | 0.2401 | No |
| 14 | ACSM1 | acyl-CoA synthetase medium chain family member 1 [Source:HGNC Symbol;Acc:HGNC:18049] | 8210 | -0.172 | 0.2081 | No |
| 15 | SPTLC2 | serine palmitoyltransferase long chain base subunit 2 [Source:HGNC Symbol;Acc:HGNC:11278] | 9534 | -0.277 | 0.1617 | No |
| 16 | SPTLC3 | serine palmitoyltransferase long chain base subunit 3 [Source:HGNC Symbol;Acc:HGNC:16253] | 11416 | -0.489 | 0.1152 | No |
Table: GSEA details [plain text format]

  

Fig 2: GOMF\_C\_ACYLTRANSFERASE\_ACTIVITY      
 Blue-Pink O' Gram in the Space of the Analyzed GeneSet

  

Fig 3: GOMF\_C\_ACYLTRANSFERASE\_ACTIVITY: Random ES distribution      
 Gene set null distribution of ES for **GOMF\_C\_ACYLTRANSFERASE\_ACTIVITY**
